# Acceptability trial of local Indonesian snack (SISTIK) enriched with chicken liver and eggshell powder as a potential food to increase micronutrient intakes among women of reproductive age

**DOI:** 10.12688/wellcomeopenres.20292.1

**Published:** 2024-09-26

**Authors:** Yenni Zuhairini, Aghnia Husnayiani Suryanto, Qorinah Estiningtyas Sakilah Adnani, Mohammad Brachim Anshari, Haidar Rizqi, Annisha Fathonah, Afini Dwi Purnamasari, Afiyah Hadiyanti Pangasih, Ayunda Jihadillah, Dina Novtyana Puspita, Dimas Erlangga Luftimas, Sofa Rahmannia, Umi Fahmida, Rosalind Gibson, Aly Diana

**Affiliations:** 1 Department of Public Health, Faculty of Medicine, Universitas Padjadjaran, Bandung, West Java, Indonesia; 2 Nutrition Working Group, Faculty of Medicine, Universitas Padjadjaran, Bandung, West Java, Indonesia; 3 Faculty of Medicine, Universitas Pasundan, Bandung, West Java, Indonesia; 4 Southeast Asian Ministers of Education Organization Regional Centre for Food and Nutrition, Jakarta, Special Capital Region of Jakarta, Indonesia; 5 Department of Nutrition, Faculty of Medicine, Universitas Indonesia, Depok, West Java, Indonesia; 6 Department of Human Nutrition, University of Otago, Dunedin, Otago, New Zealand

**Keywords:** acceptability, chicken liver, enriched food, sensory characteristics, women

## Abstract

**Background:**

Addressing stunting is a key global nutrition goal for 2025, with Indonesia among the top five countries grappling with high stunting rates in children. Chronic micronutrient deficiencies in women and young children in Indonesia have been associated with poor foetal and infant growth. To tackle this issue, we developed micronutrient-enriched crackers (MEC) incorporating nutrient-rich chicken liver and powdered eggshells. These crackers, known locally as ' *sistik*,' may provide a sustainable solution to boost micronutrient intakes. Our study among Indonesian women aimed to gauge their acceptability of MEC, which have the potential to enhance maternal micronutrient status and thus combat stunting during early childhood.

**Methods:**

We conducted a two-phase acceptability trial involving 81 women aged 19-35 years in Ujung Berung Sub-district, Bandung City, Indonesia. Each phase was a single-blinded trial; only the researcher was aware of product assignment. The first phase entailed a test feeding session in a local community house which participants sampled both MEC and standard wheat crackers (SWC) on one day. Participants assessed each product using a 7-point cued facial response scale, evaluating colour, smell, flavour, and texture. In the second phase, all participants received a 14-day supply (75 g/day) of either MEC (n=41) or SWC (n=40) to consume at home under real-life conditions. Adherence was determined by weighing unconsumed products.

**Results:**

The test MEC food received favourable ratings from participants on a 7-point scale, with no significant differences in liking scores between MEC and SWC regarding colour, smell, flavour, and texture. Mean (SD) daily adherence was 51 (21) g/d, with no significant difference between groups.

**Conclusions:**

This study provides valuable insights for stakeholders and policymakers regarding the potential options for MEC as a food or as daily snacks to increase the intakes and status of micronutrients among adult women.

**Registration:**

ClinicalTrials.gov ( NCT04564222, 25 ^th^ September 2020).

## Plain Language Summary

In Indonesia, addressing the pressing issue of child stunting is imperative due to high prevalence rates. The underlying problem of micronutrient deficiencies calls for innovative solutions. In response, we developed micronutrient-enriched crackers (MEC), or 'sistik,' enriched with chicken liver and powdered eggshells, aiming to improve maternal micronutrient status and combat childhood stunting. Our study focused on assessing MEC's acceptance among Indonesian women. To achieve this, we conducted a trial in Bandung City, involving 81 participants, to evaluate MEC's acceptance compared to standard wheat crackers (SWC). Participants reported positive feedback on MEC, appreciating its color, aroma, flavor, and texture. Furthermore, daily compliance with MEC was similar to that with SWC, indicating MEC's potential to enhance micronutrient consumption among adult women. Despite these promising results, our study recognizes limitations, including a modest sample size that may limit broader applicability of our findings. This research highlights MEC's potential to increase micronutrient intake among women, providing insights for policymakers and healthcare stakeholders. It emphasizes the need for further investigation into MEC's long-term impacts and to include a broader range of participants to ensure results are representative of adult Indonesian women.

## Introduction

Reducing the stunting rate is a primary objective of the global nutrition targets for 2025 ( WHO, 2014). Indonesia is a country with the fifth highest burden of stunted children in the world ( UNICEF, 2013). However, in the last decade in Indonesia, progress in reducing stunting has been slow, prompting the government in 2017 to prioritise national programs to combat the persistent stunting. Multiple factors have been identified as potential causes of growth retardation during infancy and early childhood in Indonesia ( Beal *et al*., 2018; Mulyaningsih *et al*., 2021; Titaley *et al*., 2019) and elsewhere ( Chilengi *et al*., 2019; Wali *et al*., 2020). Of these, chronic micronutrient deficits in the diets and biomarkers of women ( Fahmida *et al*., 2022; Rahmannia *et al*., 2019) and young children ( Diana *et al*., 2021; Fahmida *et al*., 2022), such as have been highlighted as potential deficiencies associated with poor foetal and infant linear growth. Consequently, efforts have been made to encourage the consumption of animal-source foods, a rich source of almost all the micronutrients known to play an important role in foetal growth and development ( Diana *et al*., 2021; Fahmida *et al*., 2022; Rahmannia *et al*., 2019).

Of the animal source foods, organ meats such as chicken liver have been highlighted as a potential affordable micronutrient-rich food source in Indonesia ( Fahmida *et al*., 2022; Rahmannia *et al*., 2019). Moreover, chicken liver is readily available, acceptable, and safe, although in rural settings is limited by a short shelf life. Consequently, to extend the shelf life, we chose crackers, one of favourite snack in Indonesia, as our food vehicle for enrichment with chicken livers. The type of crackers that we developed are known as "cheese sticks," called locally ' *sistik'.* However, because enrichment with chicken liver alone will not alleviate the well-established shortfall in calcium that has been identified in Indonesian diets ( Agustina *et al*., 2023; Aji *et al*., 2019), we also included powdered eggshells as an additional ingredient in the crackers. Powdered eggshells are a source of bioavailable calcium and also contain an insulin-like growth factor (IGF-1) reported to promote foetal and linear growth ( Hawkes & Grimberg, 2015; Schaafsma *et al*., 2000). Hence, together, the micronutrient-enriched crackers (MEC) will provide a rich-source of growth-promoting micronutrients (MN) with the potential to overcome the MN deficits identified in the diets of both mothers and infants in the district of our proposed study. Furthermore, the technology used to formulate the MEC is readily transferable through a local industry partner, ensuring the crackers are produced locally, are sustainable, and require no cold storage in our study setting. In this study, our primary objectives were to evaluate the acceptability of our MEC made from chicken liver and eggshell powder among Indonesian women of reproductive age; and to design a randomized controlled trial to test the effectiveness of a supplementary food, 'SISTIK', as a strategy for preventing micronutrient deficiencies.

## Methods

### Study setting, participants, and design

This research was conducted in the Ujung Berung sub-district, Bandung City, West Java, Indonesia, over a 3-month period in 2020. Ujung Berung sub-district covers an area of approximately 66.1 hectares and has a population of around 86,000 of whom 11% are engaged in crop farming. The climate is tropical, with rainfall occurring most months and only a short dry season ( BPS, 2020).

The participants were recruited by health cadres in collaboration with the Ujung Berung Indah and Pasirjati Primary Health Centre ( *Puskesmas*) from eight villages located in Ujung Berung sub-district. These villages were randomly divided into two groups: a micronutrient-enriched crackers (MEC) group and a standard wheat crackers (SWC) group. The simple randomization method was employed to assign the villages to each group, ensuring unbiased distribution. Women in every village were selected purposively due to their strategic location, ease of access, and their interest in participating in this study. Women were eligible if they were aged 19–35 years old, not pregnant, and had no health concerns that might affect their appetite, smell, or sight in the last seven days prior to enrolment. Based on ethical committee suggestions, pregnant women were excluded from the study due to their classification as a vulnerable group, in whom ethical standards aim to protect their and their unborn children's well-being, thus avoiding any potential risks associated with research participation. Women with a mid-upper arm circumference < 23.5 cm, measured with a fiberglass insertion tape (SECA 212, Germany) following standard anthropometric procedures were excluded as this threshold indicative of severe undernutrition,

### Ethical approval

Ethical clearance for this study was obtained from the Health Research Ethics Committees of Universitas Padjadjaran, Bandung, Indonesia (Approval No. 988/UN6.KEP/EC/2020, dated 19 ^th^ October 2020). The study was conducted in accordance with the ethical principles outlined in the Declaration of Helsinki. The committee's role was to protect the rights and welfare of the research subjects, ensuring that the research adhered to ethical, legal, and social implications, as well as other applicable regulations. Informed written consent was obtained from all participants prior to their inclusion in the study. They were all thoroughly informed about the study's objectives, methods, potential benefits, and risks, and assured of their right to withdraw from the study at any time without any consequences. The privacy and confidentiality of the participants were strictly maintained in accordance with applicable laws. This acceptability trial was conducted prior to the cluster randomised clinical trial, titled "Sustainable Intervention of Supplementation to Improve Kid's Growth Study (SISTIK-G Study), registered under NCT04564222 on 25th September 2020 ( https://clinicaltrials.gov/ct2/show/NCT04564222).

### Preparation of Micronutrient-Enriched Crackers (MEC) and Standard Wheat Crackers (SWC)

The recipe was developed in collaboration with the Department of Food Science at the University of Otago, Dunedin, New Zealand, and the Nutrition Working Group at *Universitas Padjadjaran*, Bandung, Indonesia, using local ingredients. To prepare MEC, raw chicken liver was first soaked in lemon juice for 15 minutes. Next, the chicken liver was boiled for 5 minutes and marinated with local herbs for 10 minutes to reduce both the liver odour and taste. Eggshells were thoroughly cleaned under running water, and their epidermal layers were removed. Afterwards, the cleaned eggshells were sun-dried for 6-8 hours, boiled for 15 minutes, and dried again in an oven (OVL-12 SS, Agrowindo, Bandung, Indonesia) for 12 hours at 60°C. Following the drying process, the eggshells were milled into a fine powder using a dry food grinder (FGD-Z300, Fomac, Jakarta, Indonesia). Finally, the chicken liver and eggshell powder were mixed with wheat flour (~74% extraction rate), tapioca flour, whole chicken eggs, salt, mushroom bouillon, margarine, seasoning, and selected herbs to form a stiff dough. SWC were created to replicate the *'sistik'* available in local markets in Indonesia. They contain wheat flour, tapioca flour, modified cassava flour (mocaf), whole chicken eggs, salt, chicken stock powder, margarine, with the addition of some herbs. To mimic the dark brown colour of MEC, rowal ( *Pangium edule*), known as *kluwak* in Indonesia, was used as a colouring agent for SWC.

The wheat flour in Indonesia used as an ingredient of both MEC and SWC is mandatory fortified with thiamin (2.5 mg/kg), riboflavin (4 mg/kg), iron (50 mg/kg), zinc (30 mg/kg), and folic acid (2 mg/kg) ( *Keputusan Menteri Kesehatan Republik Indonesia Nomor 1452/Menkes/SK/X/2003 tentang Fortifikasi Tepung Terigu*, 2003).

The stiff doughs from MEC and SWC were cut into sticks (8 cm long and 0.3 mm thick) using a pasta maker MKS-160SS (Maksindo, Malang, Indonesia) and then deep fried in palm oil. All products were packaged in sealed plastic packaging and stored at a temperature of 20–25°C and a humidity of 60–80%, reflecting the conditions commonly found in houses in West Java. The manufacturing processes for MEC and SWC, including the handling of the chicken liver and the production of the eggshells, were undertaken in accordance with a HACCP (hazard analysis critical control point) food safety plan and carried out with good manufacturing practices (GMP), following standard hygiene protocols and the Indonesia Food and Drug Supervisory Agency (BPOM) regulations (# 21 for the Food Category) ( BPOM, 2016).

The final products were analysed for macronutrients by proximate analysis, micronutrients, heavy metals (Pb, Hg, Cd, As, Sn), and microbial contamination by an accredited laboratory (Saraswati Indo Genetech Laboratory, Bogor, Indonesia (KAN LP-184-IDN with SNI ISO/IEC 17025: 2008)). Both products were tested for their shelf-life (i.e., ~12 months) following storage in sealed plastic packaging at 23–27°C (Food Technology Laboratory, *Universitas Pasundan* Bandung, Indonesia). The results of the analyses for both products are shown in Table 1. The microbiological tests showed that both MEC and SWC were within safe limits for *Salmonella sp., Bacillus cereus, Enterobacteriaceae, and Coagulase positive staphylococci* ( BPOM, 2012).

**Table 1.** Nutritional content of micronutrient-enriched crackers (MEC) and standard wheat crackers (SWC) (per 100 gram), and contamination assessment.

Components	MEC	SWC
**Macronutrients ^a^**		
Protein (%)	12.90	6.15
Fat (%)	40.44	40.04
Carbohydrate (%)	39.51	48.49
Cholesterol (mg)	142.88	18.77
Total energy (kcal)	573.58	578.86
**Micronutrients**		
Vitamin A Retinol (µg RAE) ^a^	418.39	17.69
Thiamine (mg) ^b^	0.36	0.31
Riboflavin (mg) ^b^	0.59	0.25
Niacin (mg) ^b^	4.93	3.2
Vitamin B6 (mg) ^b^	0.24	0.12
Folate (µg) ^b^	100	62.8
Vitamin B12 (µg) ^b^	3.09	0.11
Calcium (mg) ^a^	741.26	246.77
Iron (mg) ^a^	4.85	4.25
Zinc (mg) ^a^	5.93	5.19
Sodium (mg) ^a^	444.01	538.45
**Chemical and biological contamination analyses ^a^**		
Pb	Not detected	Not detected
Hg	Not detected	Not detected
Cd	Not detected	Not detected
As	Not detected	Not detected
Sn	Not detected	Not detected
*Salmonella sp.*	Negative	Negative
*Bacillus cereus*	<10	<10
*Enterobacteriaceae*	0	0
*Coagulase positive * *staphylococci*	<10	<10

^a^Laboratory analysis ^b^Calculated based on recipe

The greater amount of protein and calcium in MEC compared with SWC is due to the inclusion of chicken livers and powdered eggshells in MEC, both absent in SWC. The iron and zinc content in both products is similar, mainly because both formulations use mandatory iron- and zinc-fortified wheat flour. Additionally, the SWC product includes mocaf, which has an iron content of 15.8 mg/100 g ( Kementerian Kesehatan RI, 2020). However, the bioavailability of iron from the chicken livers in MEC is expected to be higher than that of the iron in SWC in view of the high heme iron content of liver ( Rangan *et al*., 1997). Likewise, the content of certain vitamins, notably vitamin B12 and vitamin A was also higher due to the presence of chicken liver. Although the vitamin A content of chicken livers as retinol is known to be high, our analytical data confirmed that the retinol concentration in a daily serving of MEC were well below the U.S. Tolerable Upper Intake Level (UL) (i.e., 3000 Retinol Activity Equivalents (RAE)), primarily due to degradation of retinol during processing.

### Sensory acceptability testing

This study was conducted in two phases: a feeding test and a 14-day adherence test. Each phase was conducted as a single-blinded trial, with only the researcher aware of the product assignment. On the day of the feeding test, participants were screened for eligibility, after which socio-demographic data were collected from eligible participants using a pretested structured questionnaire ( DHS Programs, 2019). Next, the participants were assigned in a 1:1 ratio to receive samples of Product A and Product B (i.e., 28g of each) to assess and evaluate the sensory characteristics of each product. For the latter, participants were instructed to indicate their acceptability of the colour, smell, taste, and texture of each test product tasted using a 7-point cued facial response scale, adapted from Pelletier and Lawless (2003), which ranged from 'dislike a lot' to 'like a lot.' For each attribute, the participants were shown three faces—a smiley face labelled 'like,' a neutral face labelled 'neutral,' and a frowning face labelled 'dislike'—and asked to indicate which face best represented their liking. If a participant chose the smiley face, she was shown three additional faces with different degrees of smiling labelled as 'like a little,' 'like,' and 'like a lot’, and again asked to indicate which of these smiley faces represented her liking. Conversely, if the participant chose the frowning face, she was shown three additional faces with varying degrees of unhappiness. From these faces, the participant was asked to select the frown which best represented her degree of disliking, ranging from 'dislike a lot,' 'dislike,' and 'dislike a little'. After completing the feeding test for the first test product, participants were instructed to have a drink of water to cleanse their palate before tasting the second test food, using the procedure described above to indicate acceptability of the colour, smell, taste, and texture via the 7-point cued facial response scale.

The second phase, the 14-day adherence test, was a home-use trial in which women's adherence to consuming either MEC or SWC in the home was assessed. In this phase, respondents were divided into two groups: Group A and Group B. Depending on their group assignment, each respondent received a 14-day supply of their assigned product (i.e., either MEC or SWC) to evaluate the acceptability of each product over an extended consumption period. Participants were asked to consume 75 g/day of their assigned product in their own homes for 14 days under real-life conditions. All respondents were encouraged to consume the product as a snack rather than as a food substitute.

During the 14-day adherence test, participants were requested to maintain a daily log, indicating whether they had consumed the entire, partial, or none of the product and whether they had encountered any challenges during consumption. To evaluate adherence, a research assistant collected the packages weekly and weighed the remaining product in each package using a digital scale (Kitchen Scale RK3131, Camry Electronic Ltd., Guangdong, China). Subsequently, the daily consumption of crackers over the 14-day period was calculated from the recorded weights of the remaining products. On completion of the two phases, interviews were conducted with each participant to gather their perception of their assigned product when consumed as a daily snack, and to assess their willingness to purchase and consume their assigned product.

### Sample size estimates and statistical analysis

The sample size in each group was calculated to detect a significant difference at the 0.05 level of probability, considering a standard deviation of 1.5 in the acceptability study ( Stone & Sidel, 2004). This calculation was based on the proportion of the study product consumed during the Phase 2 home-use trial. To ensure a statistical power of 85% and account for a potential attrition rate of 7%, a sample size of 43 women of reproductive age per group was determined.

Data analysis was conducted using IBM SPSS Statistics 20 ( IBM Corp., 2011). Descriptive statistics were calculated for socio-demographic variables. In Phase 1, means and standard deviations (SD) were calculated for the respondents' liking scores regarding colour, smell, taste, and texture. The daily adherence rate was determined as the difference between the distributed and unconsumed product divided by the number of observation days and reported as the mean (SD) (g/day). Linear regression was used to identify differences in liking between MEC and SWC. Mean differences, 95% confidence intervals (CI), and P-values were calculated and presented. Differences in the daily adherence rate between the two products were tested using a t-test.

## Results

In total, 86 participants were initially enrolled in the study, and 81 participants successfully completed both phases of the trial. Table 2 provides an overview of the socio-demographic characteristics of the women. The median age (Q1, Q3) of the respondents was 26 (23, 32) years. Approximately one-third of the participants had either received no formal education or had completed only primary education, while nearly two-thirds had attained a secondary level of education. The majority of the respondents were housewives.

**Table 2.** Socio-demographic characteristics of the respondents.

Characteristic	N=86 (%)/ Mean ± SD / Median (Q1, Q3)
Participant age (y)	26 (23, 32)
Education, n (%)	
No school/primary level	29 (33.7)
Secondary level	53 (61.6)
Tertiary level	4 (4.7)
Occupation, n (%)	
Housewife	60 (69.8)
Private employee	11 (12.8)
Entrepreneur/Traders	15 (17.4)

Table 3 presents the preferences of the respondents for MEC and SWC, as indicated by their liking scores for colour, smell, flavour, and texture from the feeding test; no significant differences existed for each of these attributes. The smell and flavour liking scores, measured on a 7-point scale (mean (SD)) were: 5.6 (0.9) and 5.6 (0.9) for SWC, and 4.9 (1.5) and 4.9 (1.4) for MEC, respectively.

**Table 3.** Acceptability of standard wheat crackers (SWC) and micronutrient-enriched crackers (MEC) by participant’s attribute scores.

Assessment method ^a^	Mean (SD)	Mean difference (95% CI)	*P-value* ^b^
Colour liking SWC MEC Smell liking SWC MEC Flavour liking SWC MEC Texture liking SWC MEC	5.4 (0.9) 4.5 (1.2) 5.6 (0.9) 4.9 (1.5) 5.6 (0.9) 4.9 (1.4) 5.7 (0.8) 5.4 (1.0)	Reference 0.110 (-0.085 – 0.250) Reference -0.195 (-0.238 – 0.014) Reference -0.045 (-0.183 – 0.122) Reference -0.06 (-0.174 – 0.164)	0.329 0.081 0.690 0.954

^a^Liking data collected using a 7-point liking scale ranging from 1=dislike a lot to 7=like a lot ^b^ *P*-values are for linear regression models used to identify if differences in participant’s liking existed between the SWC and MEC

**Figure 1.**
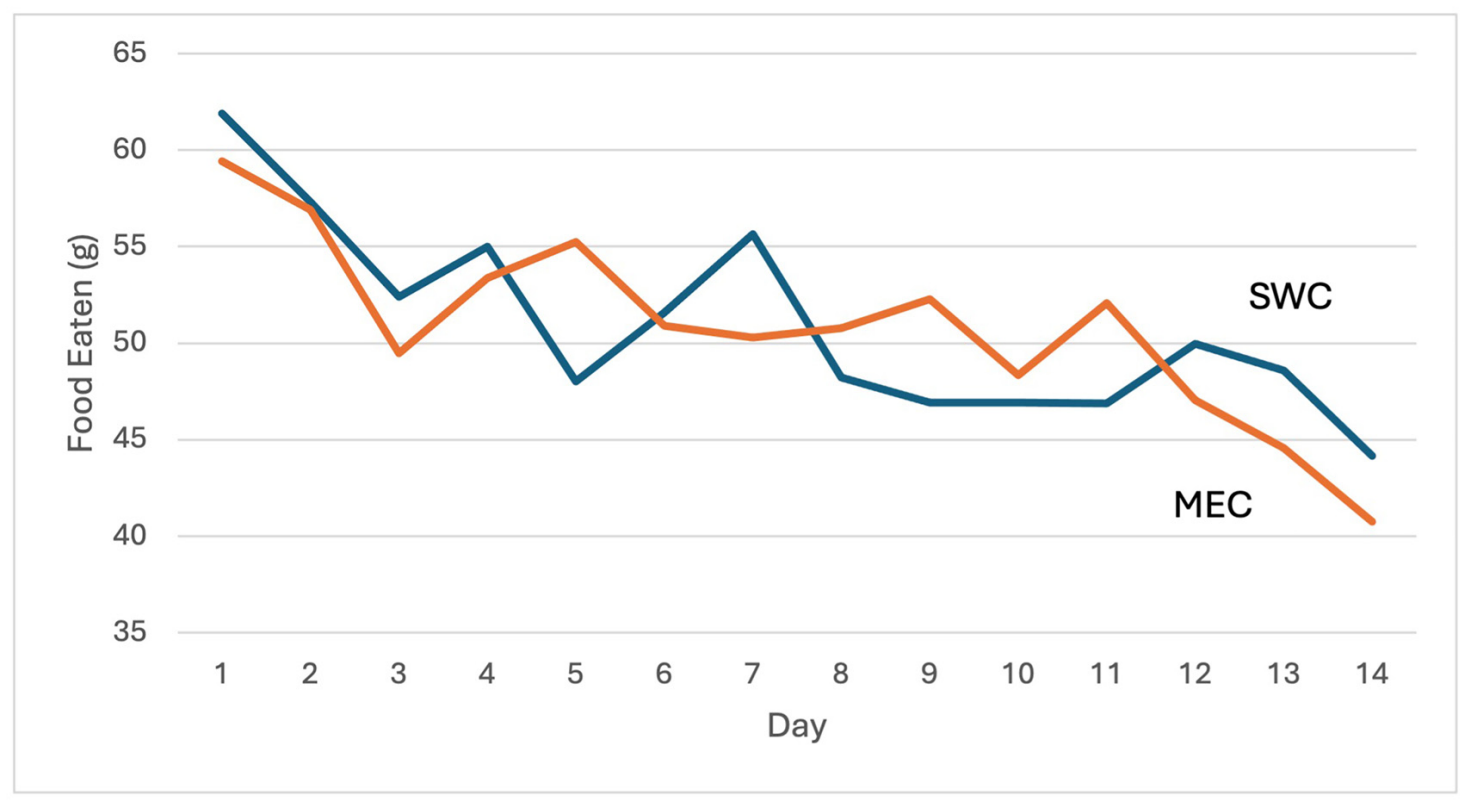
Amount of product (g/day) consumed by respondents over a 14-day period.

The adherence rates (g/day) for both MEC and SWC based on the 14-day adherence test were comparable, with participants in the MEC group consuming a mean (SD) of 50.8 (23) gr/day and those in the SWC group consuming 51.0 (20) g/day, showing no significant difference. The average daily consumption of MEC and SWC is presented in Figure 1. A similar proportion of participants achieved a considerably high level of consumption, with 44% (18 out of 41) in the MEC group and 40% (16 out of 40) in the SWC group consuming at least 80% (60 g/day) of the provided product. Furthermore, when asked about their willingness to purchase and consume the product as daily snacks, the response of most of the participants was positive.

## Discussion

This study aimed to assess the sensory acceptance of micronutrient-enriched crackers (MEC), locally known as 'sistik,' as a potential means to enhance the micronutrient adequacy of the diets of women of reproductive age in Indonesia. Our findings indicated that both MEC and SWC were acceptable to the respondents, who expressed interest in using these products as daily snacks. There were no significant differences in liking scores for colour, smell, flavour, and taste between MEC and SWC. The adherence rate was also quite promising, indicating the potential for long-term MEC consumption.

The phase 1 liking scores, while not statistically significant, hinted at a slight preference for SWC over MEC. This preference could be attributed to the more familiar texture and taste of SWC, which aligns with earlier findings in which preference for sensory attributes of familiar foods rather than those unfamiliar has been reported ( Boncinelli *et al*., 2021; Markey *et al*., 2017; Song *et al*., 2018; Stamenkovic *et al*., 2019). These findings suggest that allowing consumers time to adjust to the distinctive sensory qualities of the MEC may enhance their acceptance.

Another noteworthy observation was the comparatively lower liking score for smell associated with MEC in contrast to SWC. To address this concern, future enhancements in the MEC processing technique, such as incorporating a step of roasting the chicken liver after boiling it, may prove beneficial in mitigating the liver-like odor. Previous studies have demonstrated that meat prepared through the roasting method tends to receive significantly higher flavour ratings from panelists compared to those prepared using the steaming cooking method used here ( Alugwu & Alugwu, 2022). Exploring this alternative approach could be a worthwhile approach to improve the smell of MEC.

Our findings also indicate that a majority of respondents expressed willingness to purchase both MEC and SWC due to their perceived health benefits. Such a positive attitude is attributed to the practices in traditional communities of enhancing the nutritional quality of local foods by preparing "sistik" with eggs and adding chicken liver to local dishes. Multiple studies have demonstrated a positive impact of providing nutritional information on the acceptance of new products ( Dharni & Gupta, 2015; Jo *et al*., 2016; Plasek *et al*., 2020). Therefore, including information about the nutritional advantages of MEC as a healthy snack on the packaging may also have potential to enhance its acceptability.

Besides providing information on the nutritional quality of the product, participants also emphasized the importance of product availability and affordability when considering the purchase of these new products. Therefore, our findings suggest that the enriched crackers developed in this study could be adopted widely if the packaging was nutritionally informative, and if the product was accessible and reasonably priced in the marketplace. Certainly, earlier research has highlighted the significance of informative packaging ( Marques da Rosa *et al*., 2019; Yarar *et al*., 2019) and a higher acceptability of enriched foods when they are offered at a fair price ( Ayensu *et al*., 2019; Thompson, 2010). Therefore, future production of the MEC should consider not only the sensory attributes, but also the nutritional quality and the affordability of the MEC for women of reproductive age. These women were the target audience of this study and represent a large proportion of women from West Java and elsewhere in Indonesia.

This study provides valuable insights into the acceptability and potential benefits of MEC among women of reproductive age. One strength of our study lies in the enrichment of a commonly consumed snack with a long shelf-life, which was produced using readily available and affordable local foods and shown to enhance the micronutrient quality of the diets of women of reproductive age. In Indonesia, where research on enrichment of foods is limited, this study adds significant knowledge. However, the data collection period, although offering initial insights, was limited to 14 days, and hence does not provide a comprehensive understanding of the long-term effects of MEC consumption on micronutrient status. Future research should explore the impact of the consumption of MEC over an extended time period. Finally, the study's findings are based on a specific sample size and population, which limits the generalisability of the results. Expanding the study to include a larger, more representative sample with diverse participants would enhance the external validity of our findings.

## Conclusions

In conclusion, this study demonstrates the potential of MEC, locally known as ' *sistik*,' to enhance the micronutrient adequacy of the diets of women of reproductive age in Indonesia. Our findings reveal that both MEC and SWC were well-received by the respondents, who expressed an interest in incorporating these products into their daily snacks. Importantly, there were no significant differences in the liking scores for colour, smell, flavour, and texture between MEC and SWC. The promising adherence rate suggests the feasibility of long-term MEC consumption. However, the study highlights a preference for SWC in initial taste tests, possibly due to their familiar sensory characteristics. Future improvements in MEC processing, particularly addressing the attribute “smell”, may enhance its acceptance. Furthermore, promoting MEC's nutritional benefits and ensuring affordability and accessibility in the marketplace could further strengthen acceptability. While this study provides valuable insights, future research should explore the impact of MEC consumption over an extended time period and include more diverse participants for broader applicability of the findings.

## Ethics and consent

Ethical clearance for this study was obtained from the Health Research Ethics Committees of Universitas Padjadjaran, Bandung, Indonesia (Approval No. 988/UN6.KEP/EC/2020, dated 19 ^th^ October 2020). The study was conducted in accordance with the ethical principles outlined in the Declaration of Helsinki. The committee's role was to protect the rights and welfare of the research subjects, ensuring that the research adhered to ethical, legal, and social implications, as well as other applicable regulations. Informed written consent was obtained from all participants prior to their inclusion in the study. They were all thoroughly informed about the study's objectives, methods, potential benefits, and risks, and assured of their right to withdraw from the study at any time without any consequences.

## Data Availability

Figshare: Acceptability_Dataset.xlsx. https://doi.org/10.6084/m9.figshare.25486495 ( Diana *et al*., 2024a). Figshare: Acceptability Trial of SISTIK. https://doi.org/10.6084/m9.figshare.25486495 ( Diana *et al*., 2024b). This project includes the following extended data: Protocol SOPs Questionnaires Participant Information Sheet CONSORT checklist and diagram Data are available under the terms of the Creative Commons Attribution 4.0 International license (CC-BY 4.0).

## Software availability

The data analysis for this study was conducted using IBM SPSS Statistics 20. For those who prefer or require open-access software, we recommend using **R**, a free software environment for statistical computing and graphics, which offers equivalent functionality for descriptive statistics, linear regression, and t-tests. R is available for download at https://www.r-project.org/.
