# Acceptability trial of local Indonesian snack (SISTIK) enriched with chicken liver and eggshell powder as a potential food to increase micronutrient intakes among women of reproductive age

**DOI:** 10.12688/wellcomeopenres.20292.2

**Published:** 2025-02-24

**Authors:** Yenni Zuhairini, Aghnia Husnayiani Suryanto, Qorinah Estiningtyas Sakilah Adnani, Mohammad Brachim Anshari, Haidar Rizqi, Annisha Fathonah, Afini Dwi Purnamasari, Afiyah Hadiyanti Pangasih, Ayunda Jihadillah, Dina Novtyana Puspita, Dimas Erlangga Luftimas, Sofa Rahmannia, Umi Fahmida, Rosalind Gibson, Aly Diana

**Affiliations:** 1 Department of Public Health, Faculty of Medicine, Universitas Padjadjaran, Bandung, West Java, Indonesia; 2 Nutrition Working Group, Faculty of Medicine, Universitas Padjadjaran, Bandung, West Java, Indonesia; 3 Faculty of Medicine, Universitas Pasundan, Bandung, West Java, Indonesia; 4 Southeast Asian Ministers of Education Organization Regional Centre for Food and Nutrition, Jakarta, Special Capital Region of Jakarta, Indonesia; 5 Department of Nutrition, Faculty of Medicine, Universitas Indonesia, Depok, West Java, Indonesia; 6 Department of Human Nutrition, University of Otago, Dunedin, Otago, New Zealand

**Keywords:** acceptability, chicken liver, enriched food, sensory characteristics, women

## Abstract

**Background:**

Chronic micronutrient deficiencies in Indonesian women and young children contribute to poor foetal and infant growth. To address this, we formulated micronutrient-enriched crackers (MEC) incorporating nutrient-rich chicken liver, rich in iron, zinc, vitamin A, and B vitamins, along with powdered eggshells as a calcium source. Known locally as ' *sistik*,' MEC may provide a sustainable solution to improve micronutrient intakes. This study evaluated MEC acceptability among non-pregnant women of reproductive age to ensure safety and feasibility before extending future research to pregnant and lactating populations.

**Methods:**

A single-blinded, two-phase acceptability trial involved 81 non-pregnant women aged 19-35 years in Ujung Berung Sub-district, Bandung City, Indonesia. In Phase 1, participants sampled both MEC and standard wheat crackers (SWC) in a test feeding session and rated each product using a 7-point cued facial response scale, evaluating colour, smell, flavour, and texture. In Phase 2, participants were randomly assigned to receive a 14-day home supply (75 g/day) of either MEC (n=41) or SWC (n=40). Adherence was determined by weighing unconsumed products. Data were analysed using mixed linear model for liking scores and a t-test for adherence.

**Results:**

MEC received favourable ratings, with no significant differences compared to SWC in liking scores for colour (MEC 4.5±1.2 vs. SWC 5.4±0.9), smell (MEC 4.9±1.5 vs. SWC 5.6±0.9), flavour (MEC 4.9±1.4 vs. SWC 5.6±0.9), and texture (MEC 5.4±1.0 vs. SWC 5.7±0.8); p-value > 0.05. Average daily consumption over 14 days was comparable between groups (MEC: 50.8±23.0 g vs. SWC: 51.0±20.0 g; mean difference: -0.2 g; 95% CI: -6.5 to 6.1; p-value = 0.802).

**Conclusions:**

MEC demonstrates reasonable acceptability and feasibility as a daily snack, supporting its potential as a dietary intervention to improve women’s micronutrient intake and reduce infant stunting, especially among those women who found it appealing.

**Registration:**

ClinicalTrials.gov ( NCT04564222, 25 ^th^ September 2020).

## Plain Language Summary

In Indonesia, addressing the pressing issue of child stunting is imperative due to high prevalence rates. The underlying problem of micronutrient deficiencies calls for innovative solutions. In response, we developed micronutrient-enriched crackers (MEC), or 'sistik,' enriched with chicken liver and powdered eggshells, aiming to improve maternal micronutrient status and combat childhood stunting. Our study focused on assessing MEC's acceptance among Indonesian women. To achieve this, we conducted a trial in Bandung City, involving 81 participants, to evaluate MEC's acceptance compared to standard wheat crackers (SWC). Participants reported positive feedback on MEC, appreciating its color, aroma, flavor, and texture. Furthermore, daily compliance with MEC was similar to that with SWC, indicating MEC's potential to enhance micronutrient consumption among adult women. Despite these promising results, our study recognizes limitations, including a modest sample size that may limit broader applicability of our findings. This research highlights MEC's potential to increase micronutrient intake among women, providing insights for policymakers and healthcare stakeholders. It emphasizes the need for further investigation into MEC's long-term impacts and to include a broader range of participants to ensure results are representative of adult Indonesian women.

## Introduction

Reducing the stunting rate is a primary objective of the global nutrition targets for 2025 ( WHO, 2014). Indonesia is a country with the fifth highest burden of stunted children in the world ( UNICEF, 2013). However, in the last decade in Indonesia, progress in reducing stunting has been slow, prompting the government in 2017 to prioritise national programs to combat the persistent stunting. Multiple factors have been identified as potential causes of growth retardation during infancy and early childhood in Indonesia ( Beal *et al*., 2018; Mulyaningsih *et al*., 2021; Titaley *et al*., 2019) and elsewhere ( Chilengi *et al*., 2019; Wali *et al*., 2020). Chronic micronutrient deficits in the diets and biomarkers of pregnant and lactating women ( Fahmida *et al*., 2022; Rahmannia *et al*., 2019) and young children ( Diana *et al*., 2021; Fahmida *et al*., 2022) have been highlighted as potential deficiencies associated with poor foetal and infant linear growth, particularly deficiencies in iron, zinc, calcium, and B vitamins. Consequently, efforts have been made to encourage the consumption of animal-source foods (ASFs), a rich source of almost all the micronutrients known to play an important role in foetal growth and development ( Diana *et al*., 2021; Fahmida *et al*., 2022; Rahmannia *et al*., 2019).

Among ASFs, organ meats such as chicken liver have been highlighted as a potential affordable micronutrient-rich food source in Indonesia ( Fahmida *et al*., 2022; Rahmannia *et al*., 2019). It is particularly rich in iron, zinc, vitamin A, and B vitamins ( Table 1). Moreover, chicken liver is readily available, acceptable, and safe, although in rural settings is limited by a short shelf life. Consequently, to extend the shelf life, we chose crackers, one of favourite snack in Indonesia, as our food vehicle for enrichment with chicken livers. The type of crackers that we developed are known as "cheese sticks," called locally ' *sistik'.* However, because enrichment with chicken liver alone will not alleviate the well-established shortfall in calcium that has been identified in Indonesian diets ( Agustina *et al*., 2023; Aji *et al*., 2019), we also included powdered eggshells as an additional ingredient in the crackers. Powdered eggshells are a source of bioavailable calcium and also contain an insulin-like growth factor (IGF-1) reported to promote foetal and linear growth ( Hawkes & Grimberg, 2015; Schaafsma *et al*., 2000). Hence, together, the micronutrient-enriched crackers (MEC) will provide a rich-source of growth-promoting micronutrients (MN), including iron, zinc, calcium, vitamin A, and B vitamins, with the potential to overcome the MN deficits identified in the diets of both mothers and infants in the district of our proposed study. Furthermore, the technology used to formulate the MEC is readily transferable through a local industry partner, ensuring the crackers are produced locally, are sustainable, and require no cold storage in our study setting.

**Table 1.** Nutritional content of raw chicken liver (per 100 grams) ^a^.

Nutrient (unit)	Amount	Nutrient (unit)	Amount	Nutrient (unit)	Amount
Water (g)	76.5	Calcium (mg)	8	Vitamin C (mg)	17.9
Energy (kcal)	119	Iron (mg)	9	Thiamine (mg)	0.3
Protein (g)	16.9	Phosphorus (mg)	297	Riboflavin (mg)	1.8
Carbohydrate (g)	0.7	Potassium (mg)	230	Niacin (mg)	9.7
Fiber (g)	0	Sodium (mg)	71	Vitamin B6 (mg)	0.8
Total saturated fatty acids (g)	1.6	Zinc (mg)	2.7	Folate (µg)	588
Total MUFA (g)	1.2			Vitamin B12 (µg)	16.6
Total PUFA (g)	1.3			Vitamin A, RAE (µg)	3296
Total trans fatty acids (g)	0.1			Vitamin E (mg)	0.7
Cholesterol (mg)	345				

^a^United States Department of Agriculture, USDA (2019); https://fdc.nal.usda.gov/food-details/171060/nutrients

In this study, our primary objectives were to evaluate the acceptability of our MEC made from chicken liver and eggshell powder, specifically by evaluating liking scores (colour, smell, flavour, and texture) and 14-day adherence among Indonesian women of reproductive age. This study employed a two-sided hypothesis, testing whether significant differences existed between MEC and standard wheat crackers (SWC) in sensory acceptability and daily consumption (adherence), reflecting the exploratory nature of this research. Findings from this phase will inform future studies targeting pregnant and lactating mothers, given their critical role in improving maternal and child nutrition.

## Methods

### Study setting, participants, and design

This study was conducted in the Ujung Berung sub-district, Bandung City, West Java, Indonesia, over a 3-month period in 2020. Ujung Berung sub-district covers an area of approximately 66.1 hectares and has a population of around 86,000 of whom 11% are engaged in crop farming. The climate is tropical, with rainfall occurring most months and only a short dry season ( BPS, 2020).

A single-blinded, two-phase acceptability trial was conducted among women of reproductive age to assess sensory acceptability and adherence. In Phase 1, all participants evaluated both MEC and SWC using a 7-point cued facial response scale in a crossover design. In Phase 2, the same participants were randomly assigned to receive a 14-day supply of either MEC or SWC to assess adherence under real-life conditions. Randomization was conducted at the village level, with villages—rather than individual participants—randomly assigned to either the MEC or SWC group using simple randomization. 'MEC' and 'SWC' were written on separate pieces of paper, placed in a container, and drawn blindly by a researcher to ensure unbiased allocation.

The participants were recruited by health cadres in collaboration with the Ujung Berung Indah and Pasirjati Primary Health Centre ( *Puskesmas*) from eight villages located in Ujung Berung sub-district. Women in every village were selected purposively due to their strategic location, ease of access, and their interest in participating in this study. Strategic location’ referred to participants whose homes were located near health facilities or primary health centres, allowing for easier follow-up and monitoring. ‘Ease of access’ was determined by whether the participant’s house was reachable by vehicles, ensuring smooth transportation for both the research team and participants, as well as facilitating the delivery of study materials and follow-up visits. ‘Interest in participating’ was assessed during initial engagement sessions in which women were informed about the study objectives, and their willingness to participate was confirmed.

Women were eligible if they were aged 19–35 years old, not pregnant, and had no health concerns that might affect their appetite, smell, or sight in the last seven days prior to enrolment. Pregnant women were excluded based on ethical committee recommendations, as they are classified as a vulnerable group requiring additional protections in research. Women with a mid-upper arm circumference < 23.5 cm, measured using a fiberglass insertion tape (SECA 212, Germany) following standard anthropometric procedures were excluded as this threshold indicative of severe undernutrition

### Ethical approval

Ethical clearance for this study was obtained from the Health Research Ethics Committees of Universitas Padjadjaran, Bandung, Indonesia (Approval No. 988/UN6.KEP/EC/2020, dated 19 ^th^ October 2020). The study was conducted in accordance with the ethical principles outlined in the Declaration of Helsinki. The committee's role was to protect the rights and welfare of the research subjects, ensuring that the research adhered to ethical, legal, and social implications, as well as other applicable regulations. Informed written consent was obtained from all participants prior to their inclusion in the study. They were all thoroughly informed about the study's objectives, methods, potential benefits, and risks, and assured of their right to withdraw from the study at any time without any consequences. The privacy and confidentiality of the participants were strictly maintained in accordance with applicable laws. This acceptability trial was conducted prior to the cluster randomised clinical trial, entitled "Sustainable Intervention of Supplementation to Improve Kid's Growth Study (SISTIK-G Study), registered under NCT04564222 on 25th September 2020 ( https://clinicaltrials.gov/ct2/show/NCT04564222).

### Preparation of Micronutrient-Enriched Crackers (MEC) and Standard Wheat Crackers (SWC)

The recipe was developed in collaboration with the Department of Food Science at the University of Otago, Dunedin, New Zealand, and the Nutrition Working Group at *Universitas Padjadjaran*, Bandung, Indonesia, using local ingredients. To prepare MEC, raw chicken liver was first soaked in lemon juice for 15 minutes. Next, the chicken liver was boiled for 5 minutes and marinated with local herbs for 10 minutes to reduce both the liver odour and taste. Eggshells were thoroughly cleaned under running water, and their epidermal layers were removed. Afterwards, the cleaned eggshells were sun-dried for 6–8 hours, boiled for 15 minutes, and dried again in an oven (OVL-12 SS, Agrowindo, Bandung, Indonesia) for 12 hours at 60°C. Following the drying process, the eggshells were milled into a fine powder using a dry food grinder (FGD-Z300, Fomac, Jakarta, Indonesia). Finally, the chicken liver and eggshell powder were mixed with wheat flour (~74% extraction rate), tapioca flour, whole chicken eggs, salt, mushroom bouillon, margarine, seasoning, and selected herbs to form a stiff dough. SWC were created to replicate the *'sistik'* available in local markets in Indonesia. They contain wheat flour, tapioca flour, modified cassava flour (mocaf), whole chicken eggs, salt, chicken stock powder, margarine, with the addition of some herbs. To mimic the dark brown colour of MEC, rowal ( *Pangium edule*), known as *kluwak* in Indonesia, was used as a colouring agent for SWC.

The wheat flour in Indonesia used as an ingredient of both MEC and SWC is mandatory fortified with thiamin (2.5 mg/kg), riboflavin (4 mg/kg), iron (50 mg/kg), zinc (30 mg/kg), and folic acid (2 mg/kg) ( *Keputusan Menteri Kesehatan Republik Indonesia Nomor 1452/Menkes/SK/X/2003 tentang Fortifikasi Tepung Terigu*, 2003).

The stiff doughs from MEC and SWC were cut into sticks (8 cm long and 0.3 mm thick) using a pasta maker MKS-160SS (Maksindo, Malang, Indonesia) and then deep fried in palm oil. All products were packaged in sealed plastic packaging and stored at a temperature of 20–25°C and a humidity of 60–80%, reflecting the conditions commonly found in houses in West Java. The manufacturing processes for MEC and SWC, including the handling of the chicken liver and the production of the eggshells, were undertaken in accordance with a HACCP (hazard analysis critical control point) food safety plan and carried out with good manufacturing practices (GMP), following standard hygiene protocols and the Indonesia Food and Drug Supervisory Agency (BPOM) regulations (# 21 for the Food Category) ( BPOM, 2016).

The final products were analysed for macronutrients by proximate analysis, micronutrients, heavy metals (Pb, Hg, Cd, As, Sn), and microbial contamination by an accredited laboratory (Saraswati Indo Genetech Laboratory, Bogor, Indonesia (KAN LP-184-IDN with SNI ISO/IEC 17025: 2008)). Both products were tested for their shelf-life (i.e., ~12 months) following storage in sealed plastic packaging at 23–27°C (Food Technology Laboratory, *Universitas Pasundan* Bandung, Indonesia). The results of the analyses for both products are shown in Table 2. The microbiological tests showed that both MEC and SWC were within safe limits for *Salmonella sp., Bacillus cereus, Enterobacteriaceae, and Coagulase positive staphylococci* ( BPOM, 2012).

**Table 2.** Nutritional content of micronutrient-enriched crackers (MEC) and standard wheat crackers (SWC) (per 100 grams), and contamination assessment.

Components	MEC	SWC
**Macronutrients ^a^**		
Protein (%)	12.90	6.15
Fat (%)	40.44	40.04
Carbohydrate (%)	39.51	48.49
Cholesterol (mg)	142.88	18.77
Total energy (kcal)	573.58	578.86
**Micronutrients**		
Vitamin A Retinol (µg RAE) ^a^	418.39	17.69
Thiamine (mg) ^b^	0.36	0.31
Riboflavin (mg) ^b^	0.59	0.25
Niacin (mg) ^b^	4.93	3.2
Vitamin B6 (mg) ^b^	0.24	0.12
Folate (µg) ^b^	100	62.8
Vitamin B12 (µg) ^b^	3.09	0.11
Calcium (mg) ^a^	741.26	246.77
Iron (mg) ^a^	4.85	4.25
Zinc (mg) ^a^	5.93	5.19
Sodium (mg) ^a^	444.01	538.45
**Chemical and biological contamination analyses ^a^**		
Pb	Not detected	Not detected
Hg	Not detected	Not detected
Cd	Not detected	Not detected
As	Not detected	Not detected
Sn	Not detected	Not detected
*Salmonella sp.*	Negative	Negative
*Bacillus cereus*	<10	<10
*Enterobacteriaceae*	0	0
*Coagulase positive * *staphylococci*	<10	<10

^a^Laboratory analysis ^b^Calculated based on recipe

The greater amount of protein and calcium in MEC compared with SWC is due to the inclusion of chicken livers and powdered eggshells in MEC, both absent in SWC. The iron and zinc content in both products is similar, mainly because both formulations use mandatory iron- and zinc-fortified wheat flour. Additionally, the SWC product includes mocaf, which has an iron content of 15.8 mg/100 g ( Kementerian Kesehatan RI, 2020). However, the bioavailability of iron from the chicken livers in MEC is expected to be higher than that of the iron in SWC in view of the high heme iron content of liver ( Rangan *et al*., 1997). Likewise, the content of certain vitamins, notably vitamin B12 and vitamin A was also higher due to the presence of chicken liver. Although the vitamin A content of chicken livers as retinol is known to be high, our analytical data confirmed that the retinol concentration in a daily serving of MEC were well below the U.S. Tolerable Upper Intake Level (UL) (i.e., 3000 Retinol Activity Equivalents (RAE)), primarily due to degradation of retinol during processing.

### Sensory acceptability testing

On the day of Phase 1, participants were screened for eligibility, after which socio-demographic data were collected from eligible participants using a pretested structured questionnaire ( DHS Programs, 2019). Next, the participants were assigned in a 1:1 ratio to receive samples of MEC and SWC (i.e., 28g of each) to assess and evaluate the sensory characteristics of each product. For the latter, participants were instructed to indicate their acceptability of the colour, smell, taste, and texture of each test product tasted using a 7-point cued facial response scale, adapted from Pelletier and Lawless (2003), which ranged from 'dislike a lot' to 'like a lot.' For each attribute, the participants were shown three faces—a smiley face labelled 'like,' a neutral face labelled 'neutral,' and a frowning face labelled 'dislike'—and asked to indicate which face best represented their liking. If a participant chose the smiley face, she was shown three additional faces with different degrees of smiling labelled as 'like a little,' 'like,' and 'like a lot’, and again asked to indicate which of these smiley faces represented her liking. Conversely, if the participant chose the frowning face, she was shown three additional faces with varying degrees of unhappiness. From these faces, the participant was asked to select the frown which best represented her degree of disliking, ranging from 'dislike a lot,' 'dislike,' and 'dislike a little'. After completing Phase 1 for the first test product, participants were instructed to have a drink of water to cleanse their palate before tasting the second test food, using the procedure described above to indicate acceptability of the colour, smell, taste, and texture via the 7-point cued facial response scale.

Phase 2, the 14-day adherence test, was a home-use trial in which women's adherence to consuming either MEC or SWC in the home was assessed. Depending on their group assignment, each respondent received a 14-day supply of their assigned product (i.e., either MEC (n=41) or SWC (n=40)) to evaluate the acceptability of each product over an extended consumption period. Participants were asked to consume 75 g/day of their assigned product in their own homes for 14 days under real-life condition, with each stick weighing around 2 grams, meaning they were expected to consume approximately 35–40 sticks per day. All respondents were encouraged to consume the product as a snack rather than as a food substitute.

During Phase 2, participants were requested to maintain a daily log, indicating whether they had consumed the entire, partial, or none of the product and whether they had encountered any challenges during consumption. To evaluate adherence, a research assistant collected the packages weekly and weighed the remaining product in each package using a digital scale (Kitchen Scale RK3131, Camry Electronic Ltd., Guangdong, China). Subsequently, the daily consumption of crackers over the 14-day period was calculated from the recorded weights of the remaining products. On completion of the two phases, interviews were conducted with each participant to gather their perception of their assigned product when consumed as a daily snack, and to assess their willingness to purchase and consume their assigned product.

### Sample size estimates and statistical analysis

The sample size in each group was calculated to detect a significant difference (p<0.05) in sensory acceptability (liking) scores, considering a standard deviation (SD) of 1.5 based on previous studies ( Stone & Sidel, 2004). To achieve statistical power of 85% and account for 7% attrition, a final sample size of 43 women per group was determined.

Data analysis was conducted using IBM SPSS Statistics 20 ( IBM Corp., 2011). Descriptive statistics were calculated for socio-demographic variables. In Phase 1, means and SD were calculated for the respondents' liking scores regarding colour, smell, taste, and texture. The daily adherence rate was determined as the difference between the distributed and unconsumed product divided by the number of observation days and reported as the mean (SD) (g/day). A mixed linear model was used to determine differences in liking between MEC and SWC, with cracker type as a fixed effect and participant ID as a random effect. Mean differences, 95% confidence intervals (CI), and P-values were calculated and presented. Differences in adherence rates between the two products were tested using a t-test.

## Results

A total of 86 women were approached, of whom 81 agreed to participate and successfully completed both phases of the trial, while five were excluded due to health-related reasons. None of the respondents were lactating. Table 3 provides an overview of the socio-demographic characteristics of the women. The median age (Q1, Q3) of the respondents was 26 (23, 32) years. Approximately one-third of the participants had either received no formal education or had completed only primary education, while nearly two-thirds had attained a secondary level of education. The majority of the respondents were housewives.

**Table 3.** Socio-demographic characteristics of the respondents.

Characteristic	N=81 (%)/Mean ± SD /Median (Q1, Q3)
Participant age (y), median (Q1,Q3)	27 (23, 32)
Education, n (%)	
No school/primary level	27 (33.3)
Secondary level	50 (61.7)
Tertiary level	4 (4.9)
Occupation, n (%)	
Housewife	55 (67.9)
Private employee	11 (13.6)
Entrepreneur/Traders	15 (18.5)

Table 4 presents the preferences of the respondents for MEC and SWC, as indicated by their liking scores for colour, smell, flavour, and texture from Phase 1, measured on a 7-point scale. MEC received favourable ratings (mean±SD), with no significant differences compared to SWC in liking scores for colour (MEC 4.5±1.2 vs. SWC 5.4±0.9), smell (MEC 4.9±1.5 vs. SWC 5.6±0.9), flavour (MEC 4.9±1.4 vs. SWC 5.6±0.9), and texture (MEC 5.4±1.0 vs. SWC 5.7±0.8); p-value > 0.05.

**Table 4.** Acceptability of standard wheat crackers (SWC) and micronutrient-enriched crackers (MEC) by participant’s attribute scores.

Assessment method ^a^	N=81 (Mean (SD))	Mean difference (95% CI)	*P-value* ^b^
Colour liking SWC MEC Smell liking SWC MEC Flavour liking SWC MEC Texture liking SWC MEC	5.4 (0.9) 4.5 (1.2) 5.6 (0.9) 4.9 (1.5) 5.6 (0.9) 4.9 (1.4) 5.7 (0.8) 5.4 (1.0)	Reference 0.110 (-0.085 – 0.250) Reference -0.195 (-0.238 – 0.014) Reference -0.045 (-0.183 – 0.122) Reference -0.06 (-0.174 – 0.164)	0.329 0.081 0.690 0.954

^a^Liking data collected using a 7-point liking scale ranging from 1=dislike a lot to 7=like a lot ^b^ *P*-values are for linear regression models used to identify if differences in participant’s liking existed between the SWC and MEC

**Figure 1.**
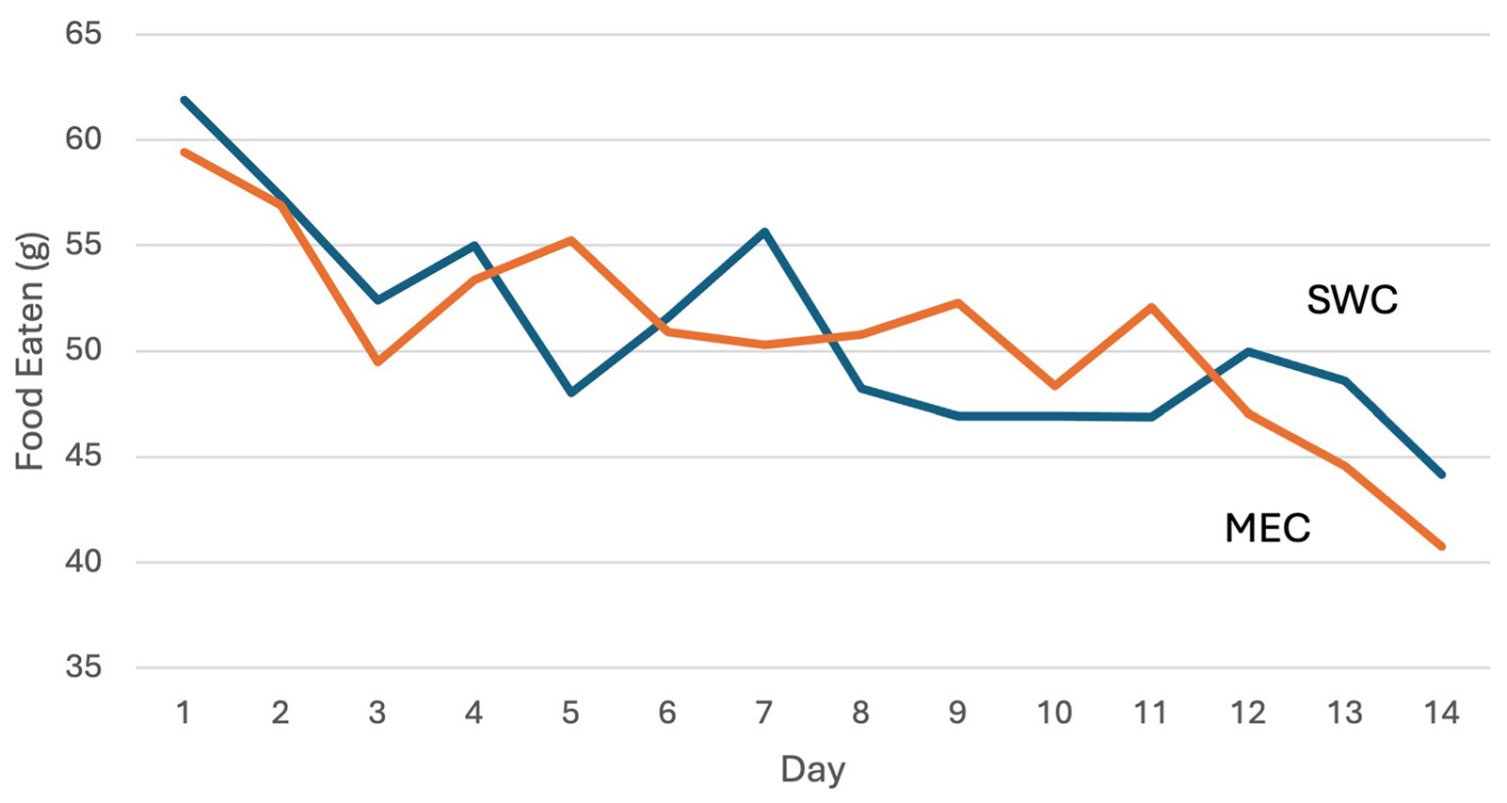
Daily consumption of the product (g/day) by respondents (n = 81) over a 14-day period.

The adherence rates (g/day) for both MEC and SWC during Phase 2 were comparable, with participants in the MEC group consuming a mean±SD of 50.8±23 gr/day and those in the SWC group consuming 51.0±20 g/day; mean difference: -0.2 g; 95% CI: -6.5 to 6.1; p = 0.802). The average daily consumption of MEC and SWC is presented in Figure 1. A significant decline in daily consumption was observed from day 1 to day 14 in both groups. In the MEC group, mean (SD) consumption decreased from 59±23 g on day 1 to 41±28 g on day 14 (p = 0.000), while in the SWC group, it declined from 62±18 g to 44±32 g (p = 0.002). The overall reduction in cracker consumption was 19±27 g for the MEC group and 18±31 g for the SWC group. Despite this decline, adherence remained relatively high among participants. Nearly half of the participants (52.5% in the MEC group and 47.5% in the SWC group) consistently consumed at least 75% of the daily portion assigned throughout the study. Furthermore, when asked about their willingness to purchase and consume the product as daily snacks, the response of most of the participants (60.5%) was positive.

## Discussion

This study aimed to assess the sensory acceptance of micronutrient-enriched crackers (MEC), locally known as ' *sistik*,' as a potential means to enhance the micronutrient adequacy of the diets of women of reproductive age in Indonesia. Our findings indicated that both MEC and SWC were acceptable to the respondents, who expressed interest in using these products as daily snacks. There were no significant differences in liking scores for colour, smell, flavour, and taste between MEC and SWC. The adherence rate was also quite promising, indicating the potential for long-term MEC consumption is feasible and primarily driven by individual preferences.

Although Phase 1 liking scores indicated a slight, non-statistically significant preference for SWC over MEC, this did not result in a long-term difference in consumption. The familiarity of SWC may have contributed to its initial appeal, which aligns with earlier findings that consumers tend to favor foods with familiar sensory attributes ( Boncinelli *et al*., 2021; Markey *et al*., 2017; Song *et al*., 2018; Stamenkovic *et al*., 2019). However, despite this initial preference, adherence rates by the end of the trial were comparable between MEC and SWC, suggesting that initial sensory preference did not translate into a long-term acceptability. While overall adherence declined over time, nearly half of the participants maintained a high level of consumption throughout Phase 2, suggesting that acceptance of MEC was influenced largely by individual preference rather than a general decline in interest. These findings suggest that allowing consumers to experience repeated exposure to MEC may help familiarize them to the distinctive sensory qualities of the MEC, potentially improving long-term acceptance among those who find it appealing.

Another noteworthy observation was the comparatively lower liking score for smell associated with MEC in contrast to SWC. To address this concern, future enhancements in the MEC processing technique, such as incorporating a step of roasting the chicken liver after boiling it, may prove beneficial in mitigating the liver-like odor. Previous studies have demonstrated that meat prepared through the roasting method tends to receive significantly higher ratings from panelists compared to those prepared using the steaming cooking method used here ( Alugwu & Alugwu, 2022). Exploring this alternative approach could be a worthwhile approach to improve the smell of MEC. Additionally, exploring alternative seasoning strategies or integrating mild aromatic ingredients may further enhance the sensory appeal of MEC without altering its nutritional composition.

Our findings also indicate that a majority of respondents expressed willingness to purchase both MEC and SWC due to their perceived health benefits. Multiple studies have demonstrated a positive impact of providing nutritional information on the acceptance of new products ( Dharni & Gupta, 2015; Jo *et al*., 2016; Plasek *et al*., 2020). In addition, earlier research has highlighted the significance of informative packaging ( Marques da Rosa *et al*., 2019; Yarar *et al*., 2019). Therefore, incorporating clear nutritional labeling on MEC packaging, including its key micronutrient benefits, could help reinforce consumer confidence and further enhance its acceptability.

Besides providing information on the nutritional quality of the product, participants also emphasized the importance of product availability and affordability when considering the purchase of these new products. Therefore, our findings suggest that the enriched crackers developed in this study could be adopted widely if the packaging was nutritionally informative, and if the product was accessible and reasonably priced in the marketplace. Previous studies show a higher acceptability of enriched foods when they are offered at a fair price ( Ayensu *et al*., 2019; Thompson, 2010). Therefore, future production of the MEC should consider not only the sensory attributes, but also the nutritional quality and the affordability of the MEC for women of reproductive age. These women were the target audience of this study and represent a large proportion of women from West Java and elsewhere in Indonesia.

This study provides valuable insights into the acceptability and potential benefits of MEC among women of reproductive age. One strength of our study lies in the enrichment of a commonly consumed snack with a long shelf-life, which was produced using readily available and affordable local foods and shown to enhance the micronutrient quality of the diets of women of reproductive age. Although iodine is essential for growth and MEC provides a small amount through its ingredients—contributing approximately 12% of the daily iodine requirement for pregnant and lactating women—this study did not aim to fortify MEC specifically with iodine. In Indonesia, iodized salt is mandated by national regulations and remains the primary dietary iodine source, making it unnecessary for the MEC to furnish the iodine requirements.

In Indonesia, where research on enrichment of foods is limited, this study adds significant knowledge. However, the data collection period, although offering initial insights, was limited to 14 days, and hence does not provide a comprehensive understanding of the long-term effects of MEC consumption on micronutrient status. Future research should explore the impact of the consumption of MEC over an extended time period on measurable improvements in key biomarkers of micronutrient status over time. Finally, the study's findings are based on a specific sample size and population, which limits the generalisability of the results. Expanding the study to include a larger, more representative sample with diverse participants would enhance the external validity of our findings.

## Conclusions

This study demonstrates the potential of MEC, locally known as ' *sistik*,' to enhance the micronutrient adequacy of the diets of women of reproductive age in Indonesia. Both MEC and SWC were well-received by the respondents, who expressed an interest in incorporating these products into their daily snacks. Importantly, there were no significant differences in the liking scores for colour, smell, flavour, and texture between MEC and SWC. The promising adherence rate suggests the feasibility of long-term MEC consumption, particularly among those who found the product appealing. Future improvements in MEC processing, particularly addressing the attribute “smell”, may enhance its acceptance. Furthermore, promoting MEC's nutritional benefits and ensuring affordability and accessibility in the marketplace could further strengthen acceptability. While this study provides valuable insights, future research should explore the impact of MEC consumption over an extended time period on micronutrient intake and status, and include more diverse participants for broader applicability of the findings.

## Ethics and consent

Ethical clearance for this study was obtained from the Health Research Ethics Committees of Universitas Padjadjaran, Bandung, Indonesia (Approval No. 988/UN6.KEP/EC/2020, dated 19 ^th^ October 2020). The study was conducted in accordance with the ethical principles outlined in the Declaration of Helsinki. The committee's role was to protect the rights and welfare of the research subjects, ensuring that the research adhered to ethical, legal, and social implications, as well as other applicable regulations. Informed written consent was obtained from all participants prior to their inclusion in the study. They were all thoroughly informed about the study's objectives, methods, potential benefits, and risks, and assured of their right to withdraw from the study at any time without any consequences.

## Data Availability

Figshare: Acceptability_Dataset.xlsx. https://doi.org/10.6084/m9.figshare.25486495 ( Diana *et al*., 2024a). Figshare: Acceptability Trial of SISTIK. https://doi.org/10.6084/m9.figshare.25486495 ( Diana *et al*., 2024b). This project includes the following extended data: Protocol SOPs Questionnaires Participant Information Sheet CONSORT checklist and diagram Data are available under the terms of the Creative Commons Attribution 4.0 International license (CC-BY 4.0).

## Software availability

The data analysis for this study was conducted using IBM SPSS Statistics 20. For those who prefer or require open-access software, we recommend using **R**, a free software environment for statistical computing and graphics, which offers equivalent functionality for descriptive statistics, linear regression, and t-tests. R is available for download at https://www.r-project.org/.
