# Supplementary material for: Acceptability trial of local Indonesian snack (SISTIK) enriched with chicken liver and eggshell powder as a potential food to increase micronutrient intakes among women of reproductive age
Source: Wellcome Open Res. 2025 Feb 24;9:550. Originally published 2024 Sep 26. [Version 2] doi: 10.12688/wellcomeopenres.20292.2 (PMC11907183; doi:10.12688/wellcomeopenres.20292.2)
Supplement: Peer review history

# Reviewer report: Reviewer response for version 2

**DOI:** 10.21956/wellcomeopenres.26317.r119557

**Published:** 2025-03-13

**Authors:** Arif Sabta Aji

**Affiliations:** 1 Health Research Organization, National Research and Innovation Agency of Indonesia, Sleman, Yogyakarta, Indonesia

We suggest the authors present a figure/picture about the SISTIK product. The specific panelist type and criteria need to be explained in detail and what the reason is to select this panelist in sensory acceptance testing. 

 It will be better if authors can transform the preparation process in workflow diagram to be more understandable.

 Authors could explain their effort to minimize the smell of product during processing as the liking score for smell is low.

 Your findings also indicate that a majority of respondents expressed willingness to purchase both MEC and SWC due to their perceived health benefits. Had participants known prior to study that they will receive two different product or a product with additional micronutrient enrich crackers?

Is the work clearly and accurately presented and does it cite the current literature?

Yes

If applicable, is the statistical analysis and its interpretation appropriate?

Yes

Are all the source data underlying the results available to ensure full reproducibility?

Yes

Is the study design appropriate and is the work technically sound?

Yes

Are the conclusions drawn adequately supported by the results?

Yes

Are sufficient details of methods and analysis provided to allow replication by others?

Yes

Reviewer Expertise:

Food and nutritional science

I confirm that I have read this submission and believe that I have an appropriate level of expertise to confirm that it is of an acceptable scientific standard, however I have significant reservations, as outlined above.

# Reviewer report: Reviewer response for version 1

**DOI:** 10.21956/wellcomeopenres.22473.r104406

**Published:** 2024-11-12

**Authors:** Alison D Gernand

**Affiliations:** 1 The Pennsylvania State University, University Park, Pennsylvania, USA

This study was a two-phase acceptability trial testing a local enriched snack compared to a standard wheat cracker. Overall the study is well conducted and described.

 I have the following major and minor comments below:

**Main comments:**

- Please enhance the results section of the abstract. Add quantitative results for the first phase (i.e., the 7-pt scale). Add the results for the difference testing (for the second phase) with a 95% CI and/or p value.
- Update the conclusion of the abstract to be a more direct/clear statement. At present, it says the study provides “valuable insights” but it would be better to specify what these are.
- In the introduction, please make a clearer, more direct connection between the opening/main concern of childhood stunting and micronutrient deficiencies in women. It may not be clear to the reader why the two are connected, and pregnancy and lactation are not mentioned.  When the authors state “potential to overcome the MN deficits identified in the diets of both mothers and infants in the district of our proposed study” – do you mean you would like to give the crackers to infants directly (in the future), or always to the mother?
- In the introduction, could you provide background or rationale behind doing the study in non-pregnant women? Did you only conduct in non-pregnant due to ethical concerns? If so, and your target is pregnant (and/or lactating) mothers, please add this. 
- When mentioning the randomized controlled trial, please specify “cluster” randomized each time (e.g. last sentence of introduction) if this is truly the study design (it is somewhat unclear - see comments below).
- In the first section of the methods (“Study setting, participants, and design”), it would be very helpful to state the design of the study. This information comes later in the methods (in “Sensory acceptability testing”) but an overall summary of the study design in the opening sentence or paragraph would be ideal for readers. It would be helpful to say in the beginning of the methods that the same participants completed both phases.
- Please provide more details on how women were selected for invitation to the study beyond: “Women in every village were selected purposively due to their strategic location, ease of access, and their interest in participating in this study.” Currently it is unclear what was considered “strategic location”; how “ease of access” was assessed, and how “interest in participating” was assessed. Add how many were approached and refused to the methods.
- In the methods, please explain how simple randomization was conducted (computer generated, etc).
- The methods state: “Each phase was conducted as a single-blinded trial…”. And earlier in the methods it states that villages were randomized into 2 groups. But in phase one, it appears that the women were not randomized but rather each participant tried both products. Similarly, phase two says “respondents were divided into two groups: Group A and Group B.” But earlier in the methods it states that the villages were randomized. Please revise and reorder the methods to correct these discrepancies.
- In the sample size description, please indicate what a “standard deviation of 1.5” is representing. Is this for a mean difference in consumption in grams? It would be helpful to state the hypothesis (in the introduction). Were you hypothesizing that there would not be a difference in consumption between MEC and SWC?
- Why did the authors use linear regression for the liking scores but a t-test for the adherence rate?
- Please consider testing the difference in consumption from day 1 to 14 (it looks like a large decline) and add this finding to the discussion. Are women getting tired of eating the product every day? Do the authors suggest this product to be eaten less than daily in the future?

**Minor comments:**

- The background in the abstract is quite long. I suggest the authors shorten it and focus more on the reasons for testing MEC in women of reproductive age (i.e. non-pregnant women) to lead into the study that was conducted.
- In the methods of the abstract, please specify that these are non-pregnant women.
- In the methods, could you clarify whether women could have been lactating?
- In the methods, please add approximately how many crackers the women were eating per day (i.e., how much is 75 g?).
- In the sample size description, the calculation is made for the “acceptability” study. However in the methods the two studies are called “feeding test” and “adherence test”). Please use the same terms throughout the study so it is clear.
- The labels on the data column of Table 2 appear to be wrong, as there are no means in the table and it says “N=86” rather than labeling n(%).
- Add the sample size to all tables and figures. If the final analytic sample size is 81, please change table 1 to represent data on the final 81 and use 81 everywhere.
- Please quantify “most” in this sentence, since the data is not in a table: “when asked about their willingness to purchase and consume the product as daily snacks, the response of most of the participants was positive.”

Is the work clearly and accurately presented and does it cite the current literature?

Partly

If applicable, is the statistical analysis and its interpretation appropriate?

Partly

Are all the source data underlying the results available to ensure full reproducibility?

Yes

Is the study design appropriate and is the work technically sound?

Partly

Are the conclusions drawn adequately supported by the results?

Yes

Are sufficient details of methods and analysis provided to allow replication by others?

Partly

Reviewer Expertise:

Nutritional sciences; maternal child nutrition; global health

I confirm that I have read this submission and believe that I have an appropriate level of expertise to confirm that it is of an acceptable scientific standard, however I have significant reservations, as outlined above.

# Response

**Published:** 2025-02-20

**Authors:** Aly Diana

**Affiliations:** SEAMEO RECFON, Indonesia

Thank you very much for your careful review and important suggestions to improve our manuscript. We really appreciate it. Below is a point-by-point response to your comments, followed by the specific changes that we have included in the revised version.

 Main comments:

- Please enhance the results section of the abstract. Add quantitative results for the first phase (i.e., the 7-pt scale). Add the results for the difference testing (for the second phase) with a 95% CI and/or p value.

**Response:** Thank you for your valuable feedback. We have revised the results section of the abstract as requested. The revised results in abstract section now reads as follows: MEC received favourable ratings, with no significant differences compared to SWC in liking scores for colour (MEC 4.5±1.2 vs. SWC 5.4±0.9), smell (MEC 4.9±1.5 vs. SWC 5.6±0.9), flavour (MEC 4.9±1.4 vs. SWC 5.6±0.9), and texture (MEC 5.4±1.0 vs. SWC 5.7±0.8); p-value > 0.05. Average daily consumption over 14 days was comparable between groups (MEC: 50.8±23.0 g vs. SWC: 51.0±20.0 g; mean difference: -0.2 g; 95% CI: -6.5 to 6.1; p-value = 0.802).

- Update the conclusion of the abstract to be a more direct/clear statement. At present, it says the study provides “valuable insights” but it would be better to specify what these are.

**Response:** Thank you for your suggestion. We have now clarified this in the conclusion of the abstract. The revised conclusions in abstract section now reads as follows: “MEC demonstrates reasonable acceptability and feasibility as a daily snack, supporting its potential as a dietary intervention to improve women’s micronutrient intake and reduce infant stunting, especially among those women who found it appealing."

- In the introduction, please make a clearer, more direct connection between the opening/main concern of childhood stunting and micronutrient deficiencies in women. It may not be clear to the reader why the two are connected, and pregnancy and lactation are not mentioned. When the authors state “potential to overcome the MN deficits identified in the diets of both mothers and infants in the district of our proposed study” – do you mean you would like to give the crackers to infants directly (in the future), or always to the mother?

**Response:** We appreciate the comment and have revised the introduction to make it clearer. Regarding the statement on overcoming MN deficits, we have clarified that the crackers will be provided to mothers, as they are to be the primary focus of the intervention. Lines in the first paragraph of Introduction now read: "Chronic micronutrient deficits in the diets and biomarkers of pregnant and lactating women (Fahmida et al., 2022; Rahmannia et al., 2019) and young children (Diana et al., 2021; Fahmida et al., 2022) have been highlighted as potential deficiencies associated with poor foetal and infant linear growth, particularly deficiencies in iron, zinc, calcium, and B vitamins." Later in the Introduction, we added: "In this study, our primary objectives were to evaluate the acceptability of MEC made from chicken liver and eggshell powder, specifically by evaluating liking scores (colour, smell, flavour, and texture) and 14-day adherence among Indonesian women of reproductive age. This study employed a two-sided hypothesis, testing whether significant differences existed between MEC and standard wheat crackers (SWC) in sensory acceptability and daily consumption (adherence), reflecting the exploratory nature of this research. Findings from this phase will inform future studies targeting pregnant and lactating mothers, given their critical role in improving maternal and child nutrition."

- In the introduction, could you provide background or rationale behind doing the study in non-pregnant women? Did you only conduct in non-pregnant due to ethical concerns? If so, and your target is pregnant (and/or lactating) mothers, please add this.

**Response:** Thank you for your suggestion. We have added this information in the Introduction (see above). We also added a statement in the Methods regarding this. Study setting, participants, and design now read: "Pregnant women were excluded based on ethical committee recommendations, as they are classified as a vulnerable group requiring additional protections in research." 

- When mentioning the randomized controlled trial, please specify “cluster” randomized each time (e.g. last sentence of introduction) if this is truly the study design (it is somewhat unclear - see comments below).

**Response:** We have checked to make sure that we included "cluster" when citing the future study: "This acceptability trial was conducted prior to the cluster randomised clinical trial, entitled "Sustainable Intervention of Supplementation to Improve Kid's Growth Study (SISTIK-G Study), registered under NCT04564222 on 25th September 2020 ( https://clinicaltrials.gov/ct2/show/NCT04564222).

- In the first section of the methods (“Study setting, participants, and design”), it would be very helpful to state the design of the study. This information comes later in the methods (in “Sensory acceptability testing”) but an overall summary of the study design in the opening sentence or paragraph would be ideal for readers. It would be helpful to say in the beginning of the methods that the same participants completed both phases.

**Response:** Thank you for your suggestion. We have revised the section of the Methods (“Study setting, participants, and design”) to include an overall summary of the study design and to clarify that the same participants completed both phases. The revised section reads as follows: "A single-blinded, two-phase acceptability trial was conducted among women of reproductive age to assess sensory acceptability and adherence. In Phase 1, all participants evaluated both MEC and SWC using a 7-point cued facial response scale in a crossover design. In Phase 2, the same participants were randomly assigned to receive a 14-day supply of either MEC or SWC to assess adherence under real-life conditions."

- Please provide more details on how women were selected for invitation to the study beyond: “Women in every village were selected purposively due to their strategic location, ease of access, and their interest in participating in this study.” Currently it is unclear what was considered “strategic location”; how “ease of access” was assessed, and how “interest in participating” was assessed. Add how many were approached and refused to the methods.

**Response:** We have revised the Methods section to clarify the selection process for participants. The definitions of "strategic location," "ease of access," and "interest in participating" have been specified, and we have included the number of women approached, those who agreed to participate, and those who were excluded. The revised text in the Methods section now reads: "Women in every village were purposively selected based on their strategic location, ease of access, and willingness to participate. ‘Strategic location’ referred to participants whose homes were located near health facilities or primary health centres, allowing for easier follow-up and monitoring. ‘Ease of access’ was determined by whether the participant’s house was reachable by vehicles, ensuring smooth transportation for both the research team and participants, as well as facilitating the delivery of study materials and follow-up visits. ‘Interest in participating’ was assessed during initial engagement sessions in which women were informed about the study objectives, and their willingness to participate was confirmed." In Results: "A total of 86 women were approached, of whom 81 agreed to participate and successfully completed both phases of the trial, while five were excluded due to health-related reasons.

- In the methods, please explain how simple randomization was conducted (computer generated, etc).

**Response:** Thank you for your comment. We have revised the Methods section to provide a clear explanation of the simple randomization process. The revised text now reads: "Randomization was conducted at the village level, with villages—rather than individual participants—randomly assigned to either the MEC or SWC group using simple randomization. 'MEC' and 'SWC' were written on separate pieces of paper, placed in a container, and drawn blindly by a researcher to ensure unbiased allocation."

- The methods state: “Each phase was conducted as a single-blinded trial…”. And earlier in the methods it states that villages were randomized into 2 groups. But in phase one, it appears that the women were not randomized but rather each participant tried both products. Similarly, phase two says “respondents were divided into two groups: Group A and Group B.” But earlier in the methods it states that the villages were randomized. Please revise and reorder the methods to correct these discrepancies.

**Response:** Thank you for your suggestion. In phase one, all participants sampled both products in a crossover design. We have clarified this in the Methods: "A single-blinded, two-phase acceptability trial was conducted among women of reproductive age to assess sensory acceptability and adherence. In Phase 1, all participants evaluated both MEC and SWC using a 7-point cued facial response scale in a crossover design. In Phase 2, participants received a 14-day supply of either MEC or SWC to assess adherence under real-life conditions. Randomization was conducted at the village level, with villages—rather than individual participants—randomly assigned to either the MEC or SWC group using simple randomization. 'MEC' and 'SWC' were written on separate pieces of paper, placed in a container, and drawn blindly by a researcher to ensure unbiased allocation."

- In the sample size description, please indicate what a “standard deviation of 1.5” is representing. Is this for a mean difference in consumption in grams? It would be helpful to state the hypothesis (in the introduction). Were you hypothesizing that there would not be a difference in consumption between MEC and SWC?

**Response:** Thank you for your valuable comment. We employed a two-sided hypothesis to assess potential differences in liking scores and daily consumption between MEC and SWC, without assuming the direction of these differences, reflecting the study’s exploratory nature. We have clarified that the standard deviation (SD) of 1.5 represents the variability in liking scores (colour, smell, flavour, and texture) from sensory testing (on a 7-point scale), used to ensure the sample size could detect meaningful differences in product acceptability. The following clarifications have been added to the manuscript: Introduction: "In this study, our primary objectives were to evaluate the acceptability of MEC made from chicken liver and eggshell powder, specifically by evaluating liking scores (colour, smell, flavour, and texture) and 14-day adherence among Indonesian women of reproductive age. This study employed a two-sided hypothesis, testing whether significant differences existed between MEC and standard wheat crackers (SWC) in sensory acceptability and daily consumption (adherence), reflecting the exploratory nature of this research. Findings from this phase will inform future studies targeting pregnant and lactating mothers, given their critical role in improving maternal and child nutrition." Methods (Sample Size): "The sample size in each group was calculated to detect a significant difference (p<0.05) in sensory acceptability (liking) scores, considering a standard deviation (SD) of 1.5 based on previous studies (Stone & Sidel, 2004). To achieve 85% power and account for 7% attrition, a final sample size of 43 women per group was determined."

- Why did the authors use linear regression for the liking scores but a t-test for the adherence rate?

***Response:*** *Thank you for your comment. A mixed linear regression model was used to analyze liking scores because each participant evaluated both MEC and SWC, making the data paired and correlated. This approach accounts for intra-individual variability by treating participant ID as a random effect, ensuring a more precise estimate of differences in liking between the two products. In contrast, a t-test was used to compare adherence rates because each participant was assigned to only one product for the 14-day home-use trial, making the data independent between groups. This distinction between paired and independent data structures guided our choice of statistical analyses, ensuring the most appropriate models were applied for each outcome.* *We have revised the Statistical Analysis section to clarify this:* A mixed linear model was used to determine mean differences in liking between MEC and SWC, with cracker type as a fixed effect and participant ID as a random effect. Mean differences, 95% confidence intervals (CI), and P-values were calculated and presented. Differences in adherence rates between the two products were tested using a t-test.

- Please consider testing the difference in consumption from day 1 to 14 (it looks like a large decline) and add this finding to the discussion. Are women getting tired of eating the product every day? Do the authors suggest this product to be eaten less than daily in the future?

**Response:** Thank you for this insightful comment. The additional analysis revealed a significant decline in cracker consumption over the 14-day period (p-value: 0.000 for MEC and p-value: 0.002 for SWC), indicating a reduction in adherence over time. This finding suggests that participants' engagement with the product decreased as the trial progressed. However, when examining actual consumption patterns, some participants fully adhered to the daily recommendation, while others exhibited moderate to lower engagement, with a noticeable decline from Day 1 to Day 14. On average, 49.4% of participants were able to consume at least 75% of the 75-gram cracker portion daily throughout the trial, with a breakdown of 52.5% in the MEC group and 47.5% in the SWC group. This variation highlights the role of individual preferences and product acceptability in sustaining adherence. In public health practice, we recognize that not every product will align with all participants' tastes, which can influence long-term adherence. To enhance the effectiveness of future intervention trials, it would be beneficial to first assess respondents' liking for the product. Targeting individuals who find the product enjoyable may help sustain adherence and maximize its impact. The following text has been added to the Results: "A significant decline in daily consumption was observed from day 1 to day 14 in both groups. In the MEC group, mean±SD consumption decreased from 59±23 g on day 1 to 41 ± 28 g on day 14 (p = 0.000), while in the SWC group, it declined from 62±18 g to 44±32 g (p = 0.002). The overall reduction was 19±27 g for the MEC group and 18±31 g for the SWC group. Despite this decline, adherence remained relatively high among participants. Nearly half of the participants (52.5% in the MEC group and 47.5% in the SWC group) consistently consumed at least 75% of the daily portion throughout the study." And the following text has been added to the Discussion : "While overall adherence declined over time, nearly half of the participants maintained a high level of consumption throughout Phase 2, suggesting that acceptance of MEC was largely influenced by individual preference rather than a general decline in interest. These findings suggest that allowing consumers with repeated exposure to MEC may help familiarize consumers with its distinctive sensory qualities, potentially improving long-term acceptance among those who find it appealing."

 Minor comments:

- The background in the abstract is quite long. I suggest the authors shorten it and focus more on the reasons for testing MEC in women of reproductive age (i.e. non-pregnant women) to lead into the study that was conducted.

**Response:** Thank you for your suggestion. We have added the reasons for testing MEC in women of reproductive age to lead into the study that was conducted. Background of the abstract now read: "Chronic micronutrient deficiencies in Indonesian women and young children contribute to poor foetal and infant growth. To address this, we formulated micronutrient-enriched crackers (MEC) incorporating nutrient-rich chicken liver, rich in iron, zinc, vitamin A, and B vitamins, along with powdered eggshells as a calcium source. Known locally as ' *sistik*,' MEC may provide a sustainable solution to improve micronutrient intakes. This study evaluated MEC acceptability among non-pregnant women of reproductive age to ensure safety and feasibility before extending future research to pregnant and lactating populations."

- In the methods of the abstract, please specify that these are non-pregnant women.

**Response:** We have added to the methods of the abstract that the participants in this study were non-pregnant women. The methods of the abstract now read: "We conducted a two-phase acceptability trial involved 81 non-pregnant women aged 19-35 in Ujung Berung Sub-district, Bandung City, Indonesia."

- In the methods, could you clarify whether women could have been lactating?

**Response:** We confirm that none of the respondents in this study were lactating at the time of data collection. Although the inclusion criteria did not explicitly exclude lactating women, we did not observe any lactating participants among those enrolled. We added this statement in the Results section: "None of the respondents were lactating."

- In the methods, please add approximately how many crackers the women were eating per day (i.e., how much is 75 g?).

**Response:** We have added this information in the Methods section: "Participants were asked to consume 75 g/day of their assigned product at home for 14 days under real-life conditions, with each stick weighing around 2 grams, meaning they were expected to consume approximately 35–40 sticks per day."

- In the sample size description, the calculation is made for the “acceptability” study. However in the methods the two studies are called “feeding test” and “adherence test”). Please use the same terms throughout the study so it is clear.

**Response:** Thank you for highlighting this inconsistency. We have carefully reviewed the manuscript and standardized the terminology to ensure consistency, using 'Phase 1' and 'Phase 2' throughout the study for clarity.

- The labels on the data column of Table 2 appear to be wrong, as there are no means in the table and it says “N=86” rather than labeling n(%).

**Response:** Thank you for your corrections. We have updated the labels and corrected the numbers in Table 3 (previously referred to as Table 2). Table 3. Socio-demographic characteristics of the respondents Characteristic N=81 (%) / Median (Q1, Q3) Participant age (y), median (Q1, Q3) 27 (23, 32) Education, n (%)      No school/primary level 27 (33.3)    Secondary level 50 (61.7)    Tertiary level 4 (4.9) Occupation, n (%)      Housewife 55 (67.9)    Private employee 11 (13.6)    Entrepreneur/Traders 15 (18.5)              

- Add the sample size to all tables and figures. If the final analytic sample size is 81, please change table 1 to represent data on the final 81 and use 81 everywhere.

**Response:** Thank you for your corrections. We have corrected the sample size to Table 3 and added sample size to Table 4 and Figure 1.

- Please quantify “most” in this sentence, since the data is not in a table: “when asked about their willingness to purchase and consume the product as daily snacks, the response of most of the participants was positive.”

**Response:** Thank you for your suggestion. We have added the quantity of participants who would purchase and consume the product as daily snacks. The last sentence in the Results now read: “Furthermore, when asked about their willingness to purchase and consume the product as daily snacks, the response of most of the participants (60.5%) was positive.”

# Reviewer report: Reviewer response for version 1

**DOI:** 10.21956/wellcomeopenres.22473.r103210

**Published:** 2024-10-15

**Authors:** Mette M Berger

**Affiliations:** 1 University of Lausanne, Lausanne, Switzerland

Addressing the important issue of stunting in their country, and aware about the high rate of micronutrient deficiencies in their population that contribute to this problem, the authors present the results of an acceptability trial involving 81 women aged 19-35 years, based on the hypothesis that correcting maternal status will impact fetal and neonatal health. The study was conducted as a single blind study comparing the intervention to standard crackers. 

 The study addresses an major issue and is well written and some shortcoming should be addressed to make improve its value.

- The abstract should provide the micronutrients targeted with the crackers (via chicken liver), mainly iron and zinc but also several B vitamins including B12 and D etc., as not all are associated with stunting (not an analytical outwork of the crackers).
- **Introduction:** Please also indicate which micronutrients you hope to influence with the crackers. Chicken liver is rich in Fe and Zn (but not only) and the addition of powdered eggshell is logical to complete calcium provision. 
- **Results: **The adherence rates (g/day) for both MEC and SWC based on the 14-day adherence test were comparable; but decreasing _fig 1! please try to explain why?
- Please provide a table with the micronutrient composition of chicken liver showing the variety of micronutrients it contains.
- Iodine is not covered with this complement but I understand it is difficult to add: Please discuss it.
- Below provision per hundred g of chicken liver 
  (https://www.nutritiontable.com/nutritions/nutrient/?id=985)

Is the work clearly and accurately presented and does it cite the current literature?

Yes

If applicable, is the statistical analysis and its interpretation appropriate?

Yes

Are all the source data underlying the results available to ensure full reproducibility?

Yes

Is the study design appropriate and is the work technically sound?

Yes

Are the conclusions drawn adequately supported by the results?

Yes

Are sufficient details of methods and analysis provided to allow replication by others?

Yes

Reviewer Expertise:

NA

I confirm that I have read this submission and believe that I have an appropriate level of expertise to confirm that it is of an acceptable scientific standard.

## References

[1] *Nutritional Information Chicken, liver* . Reference source

# Response

**Published:** 2025-02-20

**Authors:** Aly Diana

**Affiliations:** SEAMEO RECFON, Indonesia

Thank you very much for carefully reviewing our manuscript and for your constructive suggestions to improve it. *Below is a point-by-point response to your comments, followed by the specific changes that we have included in the revised version.*

- The abstract should provide the micronutrients targeted with the crackers (via chicken liver), mainly iron and zinc but also several B vitamins including B12 and D etc., as not all are associated with stunting (not an analytical outwork of the crackers).

**Response:** We appreciate your insightful suggestion. We have revised the abstract background to explicitly mention the targeted micronutrients provided by the crackers, primarily iron (Fe) and zinc (Zn), along with vitamin A and B vitamins. We have added this information in the revised Abstract, which now reads: “Chronic micronutrient deficiencies in women and young children in Indonesia have been associated with poor foetal and infant growth. To tackle this issue, we formulated micronutrient-enriched crackers (MEC) incorporating chicken liver, rich in iron, zinc, vitamin A, and B vitamins, along with powdered eggshells as a calcium source."

- Introduction: Please also indicate which micronutrients you hope to influence with the crackers. Chicken liver is rich in Fe and Zn (but not only) and the addition of powdered eggshell is logical to complete calcium provision.

**Response:** The introduction has been updated to specify the targeted micronutrients and their sources. Chicken liver is rich source of Fe, Zn, vitamin A, and B vitamins, while the addition of powdered eggshell is explained as a strategy to supplement calcium. The Introduction now reads: "Among animal source foods (ASFs), organ meats such as chicken liver have been highlighted as a potential affordable micronutrient-rich food source in Indonesia (Fahmida et al., 2022; Rahmannia et al., 2019). It is particularly rich in iron, zinc, vitamin A, and B vitamins (Table 1)."

- Results: The adherence rates (g/day) for both MEC and SWC based on the 14-day adherence test were comparable; but decreasing _fig 1! please try to explain why?

**Response:** We have added an explanation in both the Results and the Discussion. Results: "The overall reduction in cracker consumption was 19±27 g for the MEC group and 18±31 g for the SWC group. Despite this decline, adherence remained relatively high among participants. Nearly half of the participants (52.5% in the MEC group and 47.5% in the SWC group) consistently consumed at least 75% of the daily portion assigned throughout the study." Discussion:

 "While overall adherence declined over time, nearly half of the participants maintained a high level of consumption throughout Phase 2, suggesting that acceptance of MEC was influenced largely by individual preference rather than a general decline in interest. These findings suggest that allowing consumers to experience repeated exposure to MEC may help familiarize them to their distinctive sensory qualities, potentially improving long-term acceptance among those who find MEC appealing."

- Please provide a table with the micronutrient composition of chicken liver showing the variety of micronutrients it contains.

**Response:** Thank you for your input. We have added a table with the micronutrient composition per 100 grams of raw chicken liver using a source from the United States Department of Agriculture, USDA (2019). Table 1. Nutritional content of raw chicken liver (per 100 grams) ^a^ Nutrient (unit) Amount Nutrient (unit) Amount Nutrient (unit) Amount Water (g) 76.5 Calcium (mg) 8 Vitamin C (mg) 17.9 Energy (kcal) 119 Iron (mg) 9 Thiamine (mg) 0.3 Protein (g) 16.9 Phosphorus (mg) 297 Riboflavin (mg) 1.8 Carbohydrate (g) 0.7 Potassium (mg) 230 Niacin (mg) 9.7 Fiber (g) 0 Sodium (mg) 71 Vitamin B6 (mg) 0.8 Total saturated fatty acids (g) 1.6 Zinc (mg) 2.7 Folate (µg) 588 Total MUFA (g) 1.2     Vitamin B12 (µg) 16.6 Total PUFA (g) 1.3     Vitamin A, RAE (µg) 3296 Total trans fatty acids (g) 0.1 *Vitamin E (mg)* *0.7* *Cholesterol (mg)* *345* *[i] ^a^United States Department of Agriculture, USDA (2019); https://fdc.nal.usda.gov/food-details/171060/nutrients*

- *Iodine is not covered with this complement but I understand it is difficult to add: Please discuss it.*

***Response:*** *We acknowledge that iodine is a key element required for thyroid hormone production, which regulates metabolism, bone turnover, and bone mineral density, particularly during periods of growth (Inzaghi et al., 2022; Benyi & Sävendahl, 2017). Iodine deficiency is associated with iodine deficiency disorders (IDD), which can have severe health consequences, which vary according to the life stage, and may include impaired cognitive development and growth failure in children (Zimmermann & Boelaert, 2015).* *However, the primary strategy for improving iodine intake in Indonesia is the mandatory fortification of salt with potassium iodate (KIO₃). as regulated under the Indonesian National Standard (SNI 3556:2024). Given that iodized salt is widely used in households, it remains the most effective and stable source of iodine for the population (Zimmermann & Boelaert, 2015).* *Our calculation of the iodine content based on our recipe showed that MEC contains approximately 29.9 µg of iodine per pouch (75 gr), contributing around 12% of the daily iodine requirement for pregnant and lactating women. Although this contribution is modest, it is expected to complement iodine intake from other dietary sources. Nevertheless, we did not specifically target iodine in our intervention.* We have added this statement in the Limitation: Although iodine is essential for growth and MEC provides a small amount through its ingredients—contributing approximately 12% of the daily iodine requirement for pregnant and lactating women—this study did not aim to fortify MEC specifically with iodine. In Indonesia, iodized salt is mandated by national regulations and remains the primary dietary iodine source, making it unnecessary for the MEC to furnish the iodine requirements. References:

1. Inzaghi, E., Pampanini, V., Deodati, A., Cianfarani, S. The Effects of Nutrition on Linear Growth. (2022). Nutrients, 14, 1752. https://doi.org/10.3390/nu14091752
2. Benyi, E., Sävendahl, L. (2017). The Physiology of Childhood Growth: Hormonal Regulation. Hormone Research in Paediatrics, 88(1), 6–14. https://doi.org/10.1159/000471876 
3. Zimmermann, M.B.,  Boelaert, K. (2015). Iodine deficiency and thyroid disorders. The Lancet Diabetes & Endocrinology, Volume 3, Issue 4, 286-295. http://dx.doi.org/10.1016/S2213-8587(14)70225-6
4. National Standardization Agency of Indonesia (BSN). (2016). SNI 3556:2016 Garam konsumsi beriodium. National Standardization Agency of Indonesia (BSN).

- Below provision per hundred g of chicken liver

(https://www.nutritiontable.com/nutritions/nutrient/?id=985)

**Response:** We appreciate your suggestion. Previously, we used the nutritional values of raw chicken liver per 100 grams sourced from the United States Department of Agriculture (USDA). Here, we present the nutritional values of raw chicken liver per 100 grams from two sources: USDA (https://fdc.nal.usda.gov/food-details/171060/nutrients) and the Nutrition Table website (https://www.nutritiontable.com/nutritions/nutrient/?id=985). Based on the table below, the USDA provides more comprehensive micronutrient data for raw chicken liver than the Nutrition Table. Since our study focuses on micronutrient intake, we used the USDA as a reference for its more complete data. Nutrient (unit) USDA Nutrition Table Water (g) Energy (kcal) Protein (g) Carbohydrate (g) Fiber (g) Total lipid (g) Total saturated fatty acids (g) Total MUFA (g) Total PUFA (g) Total trans fatty acids (g) Cholesterol (mg) 76.5 119 16.9 0.7 0 4.8 1.6 1.2 1.3 0.1 345 71.3 132 22.5 1.0 0 4.2 1.5 1.2 1.2 - 520 Calcium (mg) Iron (mg) Phosphorus (mg) Potassium (mg) Sodium (mg) Zinc (mg) 8 9 297 230 71 2.7 12 7.5 240 260 75 3.2 Vitamin C (mg) Thiamine (mg) Riboflavin (mg) Niacin (mg) Vitamin B6 (mg) Folate (µg) Folic acid (µg) Vitamin B12 (µg) Vitamin A, RAE (µg) Retinol (mg) Vitamin E (mg) 17.9 0.3 1.8 9.7 0.8 588 0 16.6 3296 3.29 0.7 30 0.3 - - 0.8 - 2000 40 - 9.8 -                                    
